# Adventitial Cystic Disease of the Popliteal Artery with Nocturnal Rest Pain

**DOI:** 10.3390/life15020137

**Published:** 2025-01-21

**Authors:** Grigol Keshelava, Serguei Malikov

**Affiliations:** 1 Department of Vascular Surgery, Clinic Healthycore, 0112 Tbilisi, Georgia; 2 Department of Vascular Surgery, Nancy University Hospital, 54500 Nancy, France; smalikov@yahoo.com

**Keywords:** adventitial cyst, popliteal artery, rest pain

## Abstract

Arterial cystic disease (ACD) affecting the popliteal artery (PA) is a rare form of non-atherosclerotic vascular disease. This cystic tumor is defined by the accumulation of a mucinous substance in the adventitia. Treatment options include percutaneous cyst aspiration, percutaneous transluminal balloon angioplasty, the evacuation of the cyst through a surgical approach, and resection of the affected artery segment followed by arterial reconstruction using autologous venous or prosthetic grafting. Our hospital received a 36-year-old man who had an intermittent claudication and periodically nocturnal rest pain in the left lower limb. Duplex scanning and CTA showed an entrapment of the left PA by a structure related to the arterial wall with an approximate 80% stenosis. The pedal and posterior tibial pulses faded when the knee was flexed. The ACD of the PA was diagnosed. An excision of an affected arterial segment and revascularization of the PA with great saphenous vein procedures were performed. We were unable to locate any instances in the literature of ACD accompanied by nocturnal rest pain that resembled the case we have presented. At a seventeen-year follow-up, the patient’s condition was reported as normal with no intermittent claudication or rest pain in the left lower limb.

## 1. Introduction

Arterial cystic disease is a rare form of non-atherosclerotic vascular pathology. The adventitial cyst of the arteries is identified by the presence of a mucinous substance in the adventitia [1]. This disease was initially documented in 1947 by Atkins and Key in the external iliac artery [2], and only 0.1 percent of all vascular disorders are caused by this entity [3]. Intrinsic arterial compression is localized in the popliteal artery (PA) in 85% of cases; it can cause intermittent claudication or it can be asymptomatic [4]. The etiology of adventitial cystic disease (ACD) is unknown, although the following four theories have been proposed: repetitive local trauma, synovial/ganglion, systematic disorder, and embryonic development have been proposed [5]. Because none of these theories are perfect, the explanation for ACD may be more effectively described by a combination of various theories [6]. The cysts contains a mucinous fluid formed by proteoglycans, mucoproteins, mucopolysaccharids, hyaluronic acid, and hydroxyproline [7,8,9].

Physical examination reveals weakened distal pulses with knee flexion. Diagnosis is supported by ultrasonography, digital subtraction angiography, computed tomography angiography (CTA), or MRI. The differential diagnosis includes PA entrapment syndrome, chronic exertional compartment syndrome, aneurysm of the PA, atherosclerotic disease, and embolic disease [10].

The options for treatment include aspiration of the cyst, surgical excision with arterial preservation, or excision of the altered arterial segment followed by reconstruction using a vein or prosthetic graft [6,7,8,9,11]. A conservative course of treatment involves monitoring for a spontaneous resolution. Although the typical pattern is for cysts to increase in size, dissipation without intervention is possible [11].

In this study, we report a case of ACD of a left PA with a nocturnal rest pain in the lower limb. We were unable to locate any instances in the literature of ACD accompanied by nocturnal rest pain that resembled the case we have presented. We treated the patient with excision of an affected arterial segment and revascularization of the PA with the reversed great saphenous vein, which were successful and remained so for a long time.

## 2. Case Presentation

**Figure 1:**
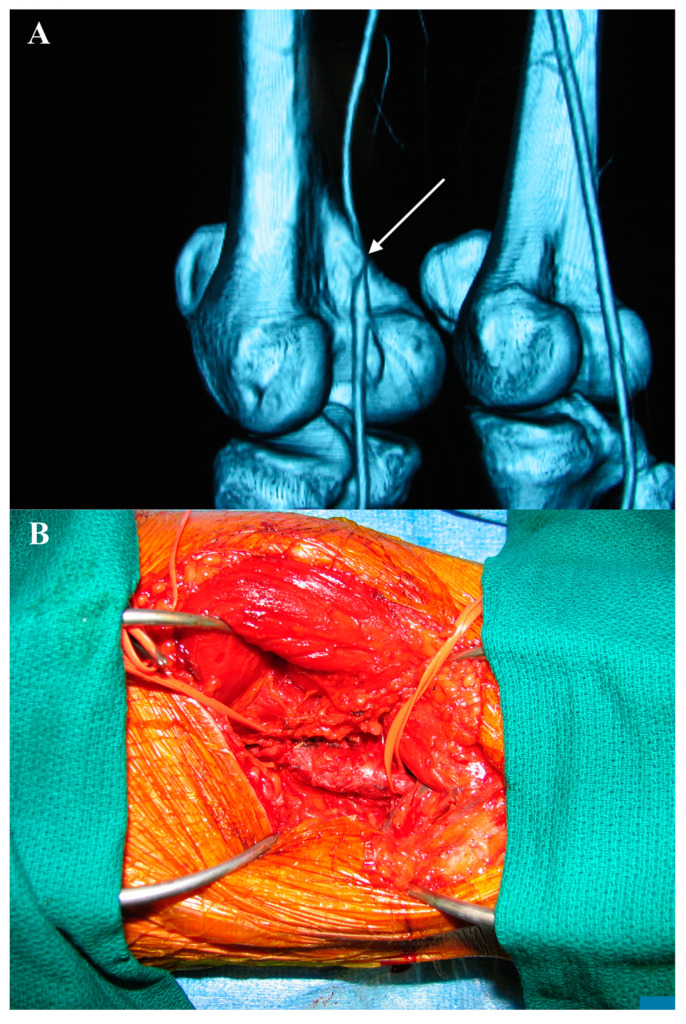
(**A**) CTA shows an entrapment of the left PA by a structure related to the arterial wall (white arrow). (**B**) The left PA is exposed by S-shaped skin incision in the popliteal fossa. An adventitial cyst is visible between the elastic loops.

Our hospital received a 36-year-old man who experienced intermittent claudication and periodically nocturnal rest pain in the left lower limb. It is noteworthy that the intermittent claudication was more pronounced than rest pain. His risk factors included tobacco abuse (one pack daily). His age at the onset of smoking was approximately 20 years old. Duplex scanning revealed severe stenosis of the left PA, and CTA showed an entrapment of the left PA by a structure related to the arterial wall with an approximate 80% stenosis of the vessel (Figure 1A). No atherosclerotic changes or obvious abnormal muscle insertion in relation to the PA were detected. The Ishikawa sign was positive: the pedal and posterior tibial pulse faded when the knee was flexed [12]. The ACD of the PA was diagnosed. In order to prevent the progression of the lower limb ischemia, the excision of an affected arterial segment and the revascularization of the PA with great saphenous vein procedures were planned. The great saphenous vein in the left limb, with a diameter of 5 mm, was marked for harvesting. The diameter of the left PA was 6 mm.

The intervention was performed on the patient under general anesthesia. A lazy S incision was made for a posterior approach to access the left popliteal fossa. Dissection was carried out between the heads of the gastrocnemius muscle to expose the PA. No anatomical abnormalities were observed in the PA in relation to the surrounding muscles and neurovascular bundle. Examination of the PA showed cystic disease in the second segment (Figure 1B). After achieving control over the healthy segments of the PA proximally and distally, the great saphenous vein was harvested from the same incision.

**Figure 2:**
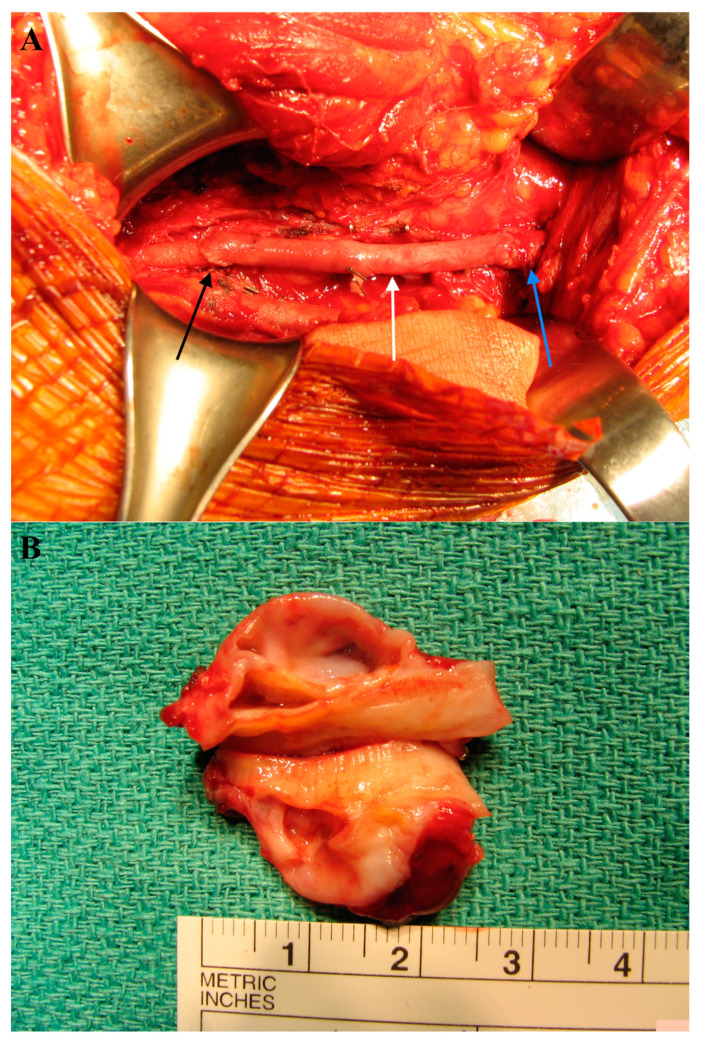
(**A**) The revascularization of the PA with an autologous reversed great saphenous vein. Blue arrow: proximal end-to-end anastomosis between the PA and venous graft; white arrow: autologous reversed great saphenous vein; black arrow: distal end-to-end anastomosis between the venous graft and PA. (**B**) Resected arterial segment with an adventitial cyst.

In the subsequent phase, following systemic heparinization (100 IU/kg) and the clamping of the proximal and distal segments of the PA, the diseased arterial segment with the adventitial cyst was resected and replaced with an autologous reversed great saphenous vein (Figure 2A). End-to-end continuous anastomosis with ‘parachuting’ was performed. A synthetic, nonabsorbable monofilament suture was performed, using an atraumatic needle (6-0 polypropylene). After revascularization, the pedal and posterior tibial pulses were present. The surgery lasted for a duration of two hours, with approximately 150 mL of blood loss recorded during the procedure. The section of the cyst demonstrated a cavitated space releasing a gelatinous substance (Figure 2B).

Under microscopic examination, the identification of a mucinous cyst degeneration of the vessel wall confirmed the presence of ACD.

Rest pain in the lower limb was not detected in the postoperative period. Distal pulses were present on clinical examination. Beginning on the first postoperative day and lasting for five days, low-molecular-weight heparin was administered. A daily dosage of 100 mg of acetylsalicylic acid was employed as a long-term antiplatelet treatment. On the fourth postoperative day, the patient was released with a prescription for 75 mg of clopidogrel and 100 mg of acetylsalicylic acid daily. At a seventeen-year follow-up, the patient’s condition was reported as normal, with no intermittent claudication or rest pain in the left lower limb. Ultrasonography revealed a patent autologous venous graft and tibial arteries.

## 3. Discussion

Several theories have been proposed regarding the origin and development of ACD: (1) the trauma theory, which posits that repetitive trauma leads to chronic degeneration; (2) the ganglion theory, which suggests that synovial cysts grow and migrate along vascular branches before settling in the adventitia of nearby major vessels; (3) the systemic disorder theory, which proposes that degeneration and cyst formation in the adventitial layer are part of a connective tissue disorder; and (4) the development theory, which proposes that mucin-secreting mesenchymal cells from nearby joints are misplaced in vessel adventitia during embryogenesis and later secrete mucin [13,14,15]. A revised developmental theory by Levien et al. suggests that it is not synovial cells, but rather undifferentiated mesenchymal cell remnants related to joints, that contribute to ACD. The authors argue that all documented cases have occurred in nonaxial vessels formed between gestational weeks 15 and 22, near developing joint structures. They propose that the concurrent development of nonaxial arteries and nearby knee, hip, wrist, or ankle joints in embryonic stages supports the hypothesis that mesenchymal tissue intended for joint formation becomes trapped in the adjacent developing nonaxial vessel. The formation of a cyst occurs later in life, when these trapped mesenchymal cell remnants begin to secrete mucoid material [15,16]. According to some authors, because none of these theories alone provide a complete explanation, ACD may be accurately understood through a synthesis of various theories [6].

Doppler ultrasonography is the primary diagnostic tool for ACD, due to its non-invasiveness. CTA and MRI enable the assessment of cyst morphology and the elimination of connections with neighboring synovial joint capsules. It is advisable to conduct three-dimensional CTA reconstructions before surgery to ensure proper surgical planning [12,17].

Typically, individuals who are young to middle-aged and in good health, especially males, are predominantly affected [18]. The typical presentation includes the onset of intermittent claudication in a lower extremity, with a longer recovery time compared to peripheral arterial disease (around 20 min) [19]. Recovery post-exertion is linked to a gradual opening of the PA due to cyst decompression [20]. Diminished distal pulses may be elicited by passive knee flexion [12]. This differs from PA entrapment syndrome, in which actions that involve contracting the gastrocnemius muscle, similar to engaging in active plantar flexion or undergoing passive dorsiflexion, lead to a decrease in pedal pulses [18,20]. Unlike the ACD, PA entrapment syndrome refers to the compression of the PA due to an abnormal anatomic relationship between the vessel and neighboring musculotendinous structures [21]. In the case of ACD, sonography reveals a narrowing of the PA, with elevated peak systolic velocity in areas of stenosis. An intramural cyst is positioned off-center to the artery, displaying low-level echoes and lacking internal flow. This imaging technique enables differentiation between ACD and a partially thrombose aneurysm, with the latter typically exhibiting a laminated thrombus and an atherosclerotic arterial wall. CTA demonstrates compression of the PA through a non-enhancing structure associated with the arterial wall, characterized by attenuation values around 40 H [22,23]. In the case of PA entrapment syndrome, sonography can reveal PA narrowing that is influenced by posture, alteration in color flow, or an elevation in peak systolic velocity. It can also assess the anatomy of the popliteal fossa and detect vascular issues such as in poststenotic aneurysm. CTA can reveal arterial stenosis and provide insights into the anatomy of the popliteal fossa [21]. Differential diagnosis should also include Baker’s cyst, which may be accompanied by pain in the knee area when flexing or extending it, in contrast to ACD [20].

All cases in the existing literature report ACD presenting with intermittent claudication. We were unable to locate any instances in the literature of ACD accompanied by nocturnal rest pain that resembled the case we have presented. In our opinion, rest pain could have developed for two reasons: first, the affected segment of the PA may have completely closed when the knee was flexed while lying down; second, when the limb was in a horizontal position, foot perfusion decreased.

A total of 90% of cases of ACD are unilateral and affect the PA. Less frequently, the external iliac, femoral, radial, ulnar, and axillary arteries may be involved [5,7,18,20]. There are rare reports of cases of ACD affecting veins [18].

Treatment options for ACD include percutaneous cyst aspiration guided by ultrasound or CTA, endovascular procedures such as percutaneous transluminal balloon angioplasty of the PA, the evacuation of the cyst through a surgical approach, and resection of the affected artery segment followed by arterial reconstruction using autologous venous or prosthetic grafting [16].

Cyst aspiration may not be feasible in cases where the cyst content is highly viscous or when the cyst is multilocular. A notable recurrence rate is observed due to the persistence of mucin-secreting mesenchymal cells, leading to fluid re-accumulation [24,25].

Endovascular treatment involving percutaneous transluminal balloon angioplasty of the affected segment of the PA is not effective for ACD, as evidenced by unsatisfactory outcomes in the existing literature. Recurrence typically occurs within 24 h to 8 weeks post-initial intervention [26,27]. The sub-adventitial cyst remains unaffected by the procedure, resulting in the reemergence of symptoms when the cyst pressure rises again, causing vessel compression. Additionally, the healthy intima of the vessel may sustain damage, increasing the risk of arterial thrombosis. While a stent-supported intervention could offer improved radial force, the placement of a stent in the PA of relatively young patients is not recommended [16]. However, the literature describes several successful cases of stenting for ACD [28,29]. According to some authors, avoiding arterial damage is possible by refraining from conducting balloon angioplasty either prior to or following stent placement [28].

Surgically opening the cyst and draining its contents is deemed a viable and effective approach to restore arterial flow in the absence of artery blockage. Removing the adventitia may offer further benefits in preventing disease recurrence. It is crucial to identify any potential link between the adventitia and the neighboring joint and to secure it with ligation [30,31].

It is advised to perform a reconstruction of the affected popliteal artery by inserting a short segment of the patient’s own vein after complete artery occlusion caused by thrombosis from cystic adventitial disease. However, numerous vascular surgeons view this method as the preferred treatment for all instances of the ACD, offering a more conclusive and secure therapeutic solution with highly favorable long-term outcomes [32]. In the case we described, autologous venous grafting showed a very successful long-term result (17 years).

## 4. Conclusions

The case described by us shows the successful long-term result of an excision of the affected artery segment and autologous reversed venous grafting for ACD with nocturnal rest pain.

## Data Availability

No new data were created or analyzed in this study.
